# Widely used but weakly studied: a research agenda for Memory Cafés

**DOI:** 10.1093/geroni/igaf143

**Published:** 2025-12-13

**Authors:** Lisa A Juckett, Kali S Thomas, Kimberly P Bernard, Sam Goodrich, Susan McFadden

**Affiliations:** School of Health and Rehabilitation Sciences, The Ohio State University, Columbus, Ohio, United States; School of Nursing, Johns Hopkins University, Baltimore, Maryland, United States; School of Public Health, Brown University, Providence, Rhode Island, United States; TimeSlips Creative Storytelling, Memory Cafe Alliance, Milwaukee, Wisconsin, United States; Department of Psychology, University of Wisconsin Oshkosh, Oshkosh, Wisconsin, United States

Originally established in the Netherlands in 1997,^1^ Memory Cafés have proliferated nationwide with over 600 Memory Cafés registered in the United States.^2^ These low-cost, community-based programs provide a safe space for people living with dementia and their care partners to socialize, learn about local supports, and engage in meaningful activities together.^3^ Memory Cafés are typically led by trained facilitators who organize events in diverse formats, including (a) joint group sessions for care partners and people with dementia, (b) peer-to-peer sessions where participants initially engage together before splitting into groups, or (c) virtual sessions via video conferencing.^4^^,^^5^ Regardless of format, Memory Cafés are widely recognized for meeting the socialization needs of both care partners and people with dementia.

Nearly three decades after their introduction, we know Memory Cafés are implementable and sustainable in community settings. Yet, research supporting their effectiveness remains limited. The current Memory Café evidence base is dominated by qualitative and non-randomized studies that consistently signal improvements in social connectedness, respite opportunities for caregivers, and quality of life.^4^^,^^6^^,^^7^ Though encouraging, these improvements are not supported by rigorous longitudinal, comparative, or causal research. Without empirical evidence, families, professionals, and aging service organizations cannot gauge the true impact of participation, which threatens continued implementation and limits access to federal, state, and charitable funding support (e.g., Older Americans Act Title III; Medicaid waivers; United Way).

Rather than developing new dementia care programs that may never be adopted, we contend that aging researchers should focus on building evidence for interventions—like Memory Cafés—that families *already* use and trust. As such, this Viewpoint article presents a *Memory Café Research Agenda* outlining three priorities: (1) defining and operationalizing Memory Café core functions, (2) rigorously evaluating participant outcomes, and (3) understanding implementation and sustainability contexts.

## Priority 1: defining and operationalizing core functions

When evaluating the effectiveness of Memory Cafés, it is essential to specify what must occur for these programs to “work.” Accordingly, the core functions of Memory Cafés should be clearly defined and supported by empirical evidence, practical experience, and stakeholder input. “Core functions” were originally described in 2006 as the essential purposes, mechanisms, or processes that drive an intervention’s effectiveness.^8^ Simply stated, an intervention will only work if all core functions are implemented as expected, and the absence of one or more core functions undermines intervention integrity. Given the ­heterogeneity of the goals of existing Memory Café programs (e.g., addressing caregiver well-being, navigating community resources, fostering social connectedness), it is likely that the primary purposes of one Memory Café may differ somewhat from those of another depending on the Café’s primary intentions and local context. While this variability presents challenges for evaluating participant outcomes, it also represents a desirable feature of the Memory Café model: its capacity to adapt to the unique strengths, needs, and preferences of each community. Studies are therefore needed to determine which elements of programming are essential to the effectiveness of Memory Café and which can be tailored to the local context and participants’ needs. Without understanding the interplay between core functions and adaptable elements, fidelity cannot be assessed, making it difficult to determine whether Memory Café programming is delivered as intended.

## Priority 2: rigorously evaluating participant outcomes

When implemented with high fidelity, Memory Cafés should yield meaningful improvements in outcomes for *both* people with dementia and their care partners; however, key questions remain about which outcomes should be prioritized based on stakeholder values and how they should be measured. Existing qualitative and non-randomized studies suggest that Memory Cafés enhance social connection, improve quality of life, and reduce stigma associated with memory loss. Yet, considerable variability in measurement approaches complicates efforts to draw firm conclusions about program effectiveness. The absence of randomized trials also limits the strength of claims that Memory Cafés directly improve social connection or mitigate dementia-related stigma. This knowledge gap underscores the need for rigorous efficacy and effectiveness trials to establish the objective value of Memory Cafés and to determine *who* benefits most and under what conditions.

## Priority 3: understanding implementation and sustainability contexts

Memory Cafés have proven implementable and sustainable in community settings, yet we have much to learn about *how* implementation approaches vary and influence participant outcomes. Accordingly, we advocate for trials that simultaneously evaluate both the effectiveness of Memory Cafés and the strategies needed to support their implementation. Given the nascent evidence base, hybrid effectiveness-implementation studies may be particularly well suited to advance the science of Memory Café programming.^9^ These hybrid studies—categorized as Type I, II, and III (Table 1)—assess the impact of Memory Cafés on participant outcomes (e.g., loneliness, sense of belonging) while *also* examining factors and strategies that influence implementation outcomes (e.g., fidelity, cost, reach).^10^ Evaluating implementation outcomes is critical for identifying the conditions under which Memory Cafés can be sustained and scaled as reliable sources of community programming. Moreover, studies should intentionally assess the outcomes and experiences of participants who represent different socioeconomic, cultural, and geographic contexts, as well as the barriers and facilitators that influence their engagement in programming. Doing so will help ensure that Memory Cafés are implemented equitably and that their benefits extend to populations that have been historically underrepresented in community-based dementia research and programs.^11^

**Table 1.** Memory café research agenda priorities and action items.

Priorities	Action items
**Priority 1: defining and operationalizing core functions**	Engage diverse stakeholders (e.g., care partners, people living with dementia, facilitators) to identify and validate core functions Develop fidelity assessment tools anchored in core functions
**Priority 2: rigorously evaluating participant outcomes**	Prioritize outcomes to measure that are meaningful to stakeholders Conduct randomized and comparative effectiveness trials to test causal pathways between memory café participation and participant outcomes Examine moderators (e.g., dementia stage, caregiver relationship) and mediators (e.g., social support) to determine who benefits most from participation in Memory Cafés and why
**Priority 3: understanding implementation and sustainability contexts**	Conduct hybrid effectiveness-implementation trials (Type I-III) Assess key implementation outcomes, including fidelity, cost, and reach Investigate the factors and strategies that influence scalability and long-term sustainability

## Conclusion

Memory Cafés are widely implemented, low-cost interventions that communities have valued for nearly 30 years. Yet, the empirical base lags behind their popularity. We urge aging researchers to build evidence for well-established dementia care interventions—starting with Memory Cafés—before creating the next new program. Addressing the proposed research priorities will strengthen families’ trust in the effectiveness of Memory Cafés and broaden our field’s understanding of how best to support their continued implementation across communities.

## Funding

None declared.

## Conflict of interest

None declared.

## Data Availability

A synthesis of Memory Café literature used to inform the direction of this manuscript is available upon reasonable request to the corresponding author.
